# EntropyHub: An open-source toolkit for entropic time series analysis

**DOI:** 10.1371/journal.pone.0259448

**Published:** 2021-11-04

**Authors:** Matthew W. Flood, Bernd Grimm

**Affiliations:** Human Motion, Orthopaedics, Sports Medicine and Digital Methods (HOSD), Luxembourg Institute of Health (LIH), Eich, Luxembourg; Shahrood University of Technology, ISLAMIC REPUBLIC OF IRAN

## Abstract

An increasing number of studies across many research fields from biomedical engineering to finance are employing measures of entropy to quantify the regularity, variability or randomness of time series and image data. Entropy, as it relates to information theory and dynamical systems theory, can be estimated in many ways, with newly developed methods being continuously introduced in the scientific literature. Despite the growing interest in entropic time series and image analysis, there is a shortage of validated, open-source software tools that enable researchers to apply these methods. To date, packages for performing entropy analysis are often run using graphical user interfaces, lack the necessary supporting documentation, or do not include functions for more advanced entropy methods, such as cross-entropy, multiscale cross-entropy or bidimensional entropy. In light of this, this paper introduces *EntropyHub*, an open-source toolkit for performing entropic time series analysis in MATLAB, Python and Julia. EntropyHub (version 0.1) provides an extensive range of more than forty functions for estimating cross-, multiscale, multiscale cross-, and bidimensional entropy, each including a number of keyword arguments that allows the user to specify multiple parameters in the entropy calculation. Instructions for installation, descriptions of function syntax, and examples of use are fully detailed in the supporting documentation, available on the EntropyHub website– www.EntropyHub.xyz. Compatible with Windows, Mac and Linux operating systems, EntropyHub is hosted on GitHub, as well as the native package repository for MATLAB, Python and Julia, respectively. The goal of EntropyHub is to integrate the many established entropy methods into one complete resource, providing tools that make advanced entropic time series analysis straightforward and reproducible.

## Introduction

Through the lens of probability, information and uncertainty can be viewed as conversely related—the more uncertainty there is, the more information we gain by removing that uncertainty. This is the principle behind Shannon’s formulation of entropy (1) which quantifies uncertainty as it pertains to random processes [1]:

$$H(X)=-\sum_{i=1}^{n}p(x_{i})\log_{b}\;p(x_{i})$$ (1)

where *H*(*X*) is the entropy (*H*) of a sequence (*X*) given the probabilities (*p*) of states (*x*_*i*_). An extension of Shannon’s entropy, conditional entropy (2) measures the information gained about a process (*X*) conditional on prior information given by a process *Y*,

$$H(X|Y)=-\sum_{i=1}^{n}p(x_{i}|Y=y)\log_{b}\;p(x_{i}|Y=y)$$ (2)

where *y* may represent states of a separate system or previous states of the same system. Numerous variants have since been derived from conditional entropy, and to a lesser extent Shannon’s entropy, to estimate the information content of time series data across various scientific domains [2], resulting in what has recently been termed “the entropy universe” [3]. This universe of entropies continues to expand as more and more methods are derived with improved statistical properties over their precursors, such as robustness to short signal lengths [4–7], resilience to noise [8–10], insensitivity to amplitude fluctuations [11–13]. Furthermore, new entropy variants are being identified which quantify the variability of time series data in specific applications, including assessments of cardiac disease from electrocardiograms [14–16], and examinations of machine failure from vibration signals [17, 18].

As the popularity of entropy spreads beyond the field of mathematics to subjects ranging from neurophysiology [19–23] to finance [24–27], there is an emerging demand for software packages with which to perform entropic time series analysis. Open-source software plays a critical role in tackling the replication crisis in science by providing validated algorithmic tools that are available to all researchers [28, 29]. Without access to these software tools, researchers lacking computer programming literacy may resort to borrowing algorithms from unverified sources which could be vulnerable to coding errors. Furthermore, software packages often serve as entry points for researchers unfamiliar with a subject to develop an understanding of the most commonly used methods and how they are applied. This point is particularly relevant in the context of entropy, a concept that is often misinterpreted [3, 30, 31], and where the name and number variant methods may be difficult to follow. For example, derivatives of the original sample entropy algorithm [32], already an improvement on approximate entropy [33], include modified sample entropy (fuzzy entropy) [34], multiscale (sample) entropy [15], composite multiscale entropy [35], refined multiscale entropy [14], and refined-composite multiscale entropy [36].

Several packages offering entropy-related functions have been released in recent years [37–39], intended primarily for the analysis of physiological data, Table 1. Although these packages offer some useful tools, they lack the capacity to perform extensive data analysis with multiple methods from the cross-entropy [40], bidimensional entropy [41], and multiscale entropy [42] families of algorithms. Additionally, the utility of these packages is also limited for several reasons. The *CEPS* [38], *EZ Entropy* [37] and *PyBioS* [39] packages all operate through graphical user interfaces (GUIs) with facilities to plot and process data interactively. The interactive nature of GUIs can be beneficial when analysing small datasets but becomes burdensome when analysing large datasets where automated processing tasks are advantageous. Both the *CEPS* [38] and *EZ Entropy* [37] are designed for the MATLAB programming environment (MathWorks, MA, USA) which requires a purchased license in order to use. This paywall prevents many users from accessing the software and consequently impedes the replication of results achieved by using these packages. Neither *PyBioS* nor *EZ Entropy* have accompanying documentation to describe how to use the software, and neither toolbox is hosted on the native package repository for MATLAB (MathWorks File Exchange) or Python (PyPi), which facilitate direct and simplified installation and updating.

**Table 1:** A list of resources providing entropy analysis tools.

Name	Language	Interface	Access Links	Details
**EntropyHub**	MATLAB	Command Line	• MATLAB Add-On Explorer • Python Package Index (PyPi) • JuliaHub • GitHub • Julia GitHub Repo • www.EntropyHub.xyz	See Table 2 for full list of functions in version 0.1. EntropyHub provides 18 *Base* entropy methods for univariate data analysis (e.g. sample entropy, fuzzy entropy, etc.), and 8 *Cross-*entropy methods (e.g. cross-permutation entropy, cross-distribution entropy). There are also 4 bidimensional entropy methods for 2D/image analysis (e.g. bidimensional dispersion entropy, bidimensional sample entropy). There are also several multiscale entropy variants available which can utilise each of the *Base* and *Cross-*entropy methods.
	Python			
	Julia			
CEPS [38]	MATLAB	GUI	BitBucket	Includes Shannon, Rényi, minimum, Tsallis, Kolmogorov-Sinai, conditional, corrected-conditional, approximate, sample, fuzzy, permutation, distribution, dispersion, phase, slope, bubble, spectral, differential, diffusion, and multiscale entropy methods.
PyBios [39]	Python	GUI	*Contact Author*	Includes sample, fuzzy, permutation, distribution, dispersion, phase, multiscale entropy methods.
EZ Entropy [37]	MATLAB	GUI	GitHub	Includes approximate, sample, fuzzy, permutation, distribution and conditional entropy methods.
PhysioNet [43]	MATLAB C*	Command Line	www.PhysioNet.org	Provides standalone functions for sample, multiscale and transfer entropies*.

Listed next to each tool are the programming languages they support, the interface through which they operate, links to access the software, and a brief outline of the entropy analysis tools they provide.

* A C-programming implementation of transfer entropy is currently not available on PhysioNet.

Against this background, this paper introduces *EntropyHub*, an open-source toolkit for entropic time series analysis in the MATLAB, Python [44] and Julia [45] programming environments. Incorporating entropy estimators from information theory, probability theory and dynamical systems theory, EntropyHub features a wide range of functions to calculate the entropy of, and the cross-entropy between, univariate time series data. In contrast to other entropy-focused toolboxes, EntropyHub runs from the command line without the use of a GUI and provides many new benefits, including:

■ Functions to perform refined, composite, refined-composite and hierarchical multiscale entropy analysis using more than twenty-five different entropy and cross-entropy estimators (approximate entropy, cross-sample entropy, etc).
■ Functions to calculate bidimensional entropies from two-dimensional (image) data.
■ An extensive range of function arguments to specify additional parameter values in the entropy calculation, including options for time-delayed state-space reconstruction and entropy value normalisation where possible.
■ Availability in multiple programming languages–MATLAB, Python, Julia–to enable open-source access and provide cross-platform translation of methods through consistent function syntax. To the best of the Authors’ knowledge, this is the first entropy-specific toolkit for the Julia language.
■ Compatible with both Windows, Mac and Linux operating systems.
■ Comprehensive documentation describing installation, function syntax, examples of use, and references to source literature. Documentation is available online at www.EntropyHub.xyz (or at MattWillFlood.github.io/EntropyHub), where it can also be downloaded as a booklet (*EntropyHub Guide*.*pdf*). Documentation specific to the MATLAB edition can also be found in the ‘supplemental software’ section of the MATLAB help browser after installation. Documentation specific to the Julia edition can also be found at MattWillFlood.github.io/EntropyHub.jl/stable.
■ Hosting on the native package repositories for MATLAB (MathWorks File Exchange), Python (PyPi) and Julia (Julia General Registry), to facilitate straightforward downloading, installation and updating. The latest development releases can also be downloaded from the EntropyHub GitHub repository - www.github.com/MattWillFlood/EntropyHub.

As new measures enter the ever-growing entropy universe, EntropyHub aims to incorporate these measures accordingly. EntropyHub is licensed under the Apache license (version 2.0) and is available for use by all on condition that the present paper by cited on any scientific outputs realised using the EntropyHub toolkit.

The following sections of the paper outline the toolkit contents, steps for installing and accessing documentation.

## Toolkit contents and functionality

Functions in the EntropyHub toolkit fall into five categories. The first three categories—*Base*, *Cross* and *Bidimensional—*refer to standalone entropy estimators distinguished according to the type of input data they analyse.

■ *Base* functions return the entropy of a single univariate time series, e.g. sample entropy (SampEn), bubble entropy (BubbEn), phase entropy (PhasEn), etc.
■ *Cross* functions return the cross-entropy *between* two univariate time series, e.g. cross-fuzzy entropy (XFuzzEn), cross-permutation entropy (XPermEn), etc.
■ *Bidimensional* functions return the entropy from a univariate, two-dimensional data matrix, e.g. bidimensional distribution entropy (DistEn2D), etc.

The remaining two categories–*Multiscale* and *Multiscale Cross–*relate to multiscale entropy methods using the entropy estimators from the *Base* and *Cross* categories, respectively.

■ *Multiscale* functions return the multiscale entropy of a single univariate time series, calculated using any of the *Base* entropy estimators,
  ■ e.g. multiscale entropy (MSEn), composite multiscale entropy (cMSEn), etc.
■ *Multiscale Cross* functions return the multiscale cross-entropy *between* two univariate time series calculated using any of the *Cross* entropy estimators,
  ■ e.g. cross-multiscale entropy (XMSEn), refined multiscale cross-entropy (rXMSEn), etc.

A list of all functions available in version 0.1 of the EntropyHub toolkit is provided in Table 2. As more entropy methods are identified, these will be added to newer versions of the toolkit.

**Table 2:** List of base, cross, bidimensional, multiscale and multiscale cross-entropy functions available in version 0.1 of the EntropyHub toolkit.

	Entropy Method	Function Name	References
**Base Entropy Functions**	Approximate Entropy	ApEn	[33]
	Attention Entropy	AttnEn	[46]
	Bubble Entropy	BubbEn	[47]
	(corrected) Conditional Entropy	CondEn	[48]
	Cosine Similarity Entropy	CoSiEn	[49]
	Dispersion Entropy	DispEn	[8, 50–52]
	Distribution Entropy	DistEn	[6]
	Entropy of Entropy	EnofEn	[53]
	Fuzzy Entropy	FuzzEn	[13, 34]
	Gridded Distribution Entropy	GridEn	[54–58]
	Increment Entropy	IncrEn	[59–61]
	Kolmogorov Entropy	K2En	[62–64]
	Permutation Entropy	PermEn	[11, 12, 65–71]
	Phase Entropy	PhasEn	[72]
	Sample Entropy	SampEn	[32]
	Slope Entropy	SlopEn	[73]
	Spectral Entropy ^†^	SpecEn	[74, 75]
	Symbolic Dynamic Entropy	SyDyEn	[76–78]
**Cross-Entropy Functions**	Cross-Approximate Entropy	XApEn	[33]
	(corrected) Cross-Conditional Entropy	XCondEn	[48]
	Cross-Distribution Entropy	XDistEn	[6, 79]
	Cross-Fuzzy Entropy	XFuzzEn	[80]
	Cross-Kolmogorov Entropy ^§^	XK2En	
	Cross-Permutation Entropy	XPermEn	[81]
	Cross-Sample Entropy	XSampEn	[32]
	Cross-Spectral Entropy ^§^	XSpecEn	
**Bidimensional Entropy Functions**	Bidimensional Distribution Entropy	DistEn2D	[82]
	Bidimensional Dispersion Entropy	DispEn2D	[83]
	Bidimensional Fuzzy Entropy	FuzzEn2D	[84, 85]
	Bidimensional Sample Entropy	SampEn2D	[86]
**Multiscale Entropy Functions**	Multiscale Entropy	MSEn	[15, 22, 87–94]
	Composite Multiscale Entropy	cMSEn	[5, 35, 36]
	(+ Refined-Composite Multiscale Entropy)		
	Refined Multiscale Entropy	rMSEn	[14, 95]
	Hierarchical Multiscale Entropy	hMSEn	[96]
**Multiscale Cross-Entropy Functions**	Multiscale Cross-Entropy	XMSEn	[15, 40, 97–100]
	Composite Multiscale Cross-Entropy	cXMSEn	[101]
	(+ Refined-Composite Multiscale Cross-Entropy)		
	Refined Multiscale Cross-Entropy	rXMSEn	[14, 101]
	Hierarchical Multiscale Cross-Entropy	hXMSEn	[96]
**Other**	Multiscale Entropy Object *	MSobject	
	Example Data Importer **	ExampleData	

* The multiscale entropy object returned by MSobject function is a required argument for *Multiscale* and *Multiscale Cross* function*s*.

** Sample time series and image data can be imported using the ExampleData function. Use of this function requires an internet connection. The imported data are the same as those used in the examples provided in the EntropyHub documentation.

^†^ In contrast to other *Base* entropies, spectral entropy (SpecEn) is not derived from information theory or dynamical systems theory, and instead measures the entropy of the frequency spectrum.

^§^ Cross-Kolmogorov and cross-spectral entropies, while included in the toolkit, have yet to be verified in the scientific literature.

One of the main advantages of EntropyHub is the ability to specify numerous parameters used in the entropy calculation by entering optional keyword function arguments. The default value of each keyword argument is based on the value proposed in the original source literature for that method. However, blindly analysing time series data using these arguments is strongly discouraged. Drawing conclusions about data based on entropy values is only valid when the parameters used to calculate those values accurately capture the underlying dynamics of the data.

**Fig 1:**
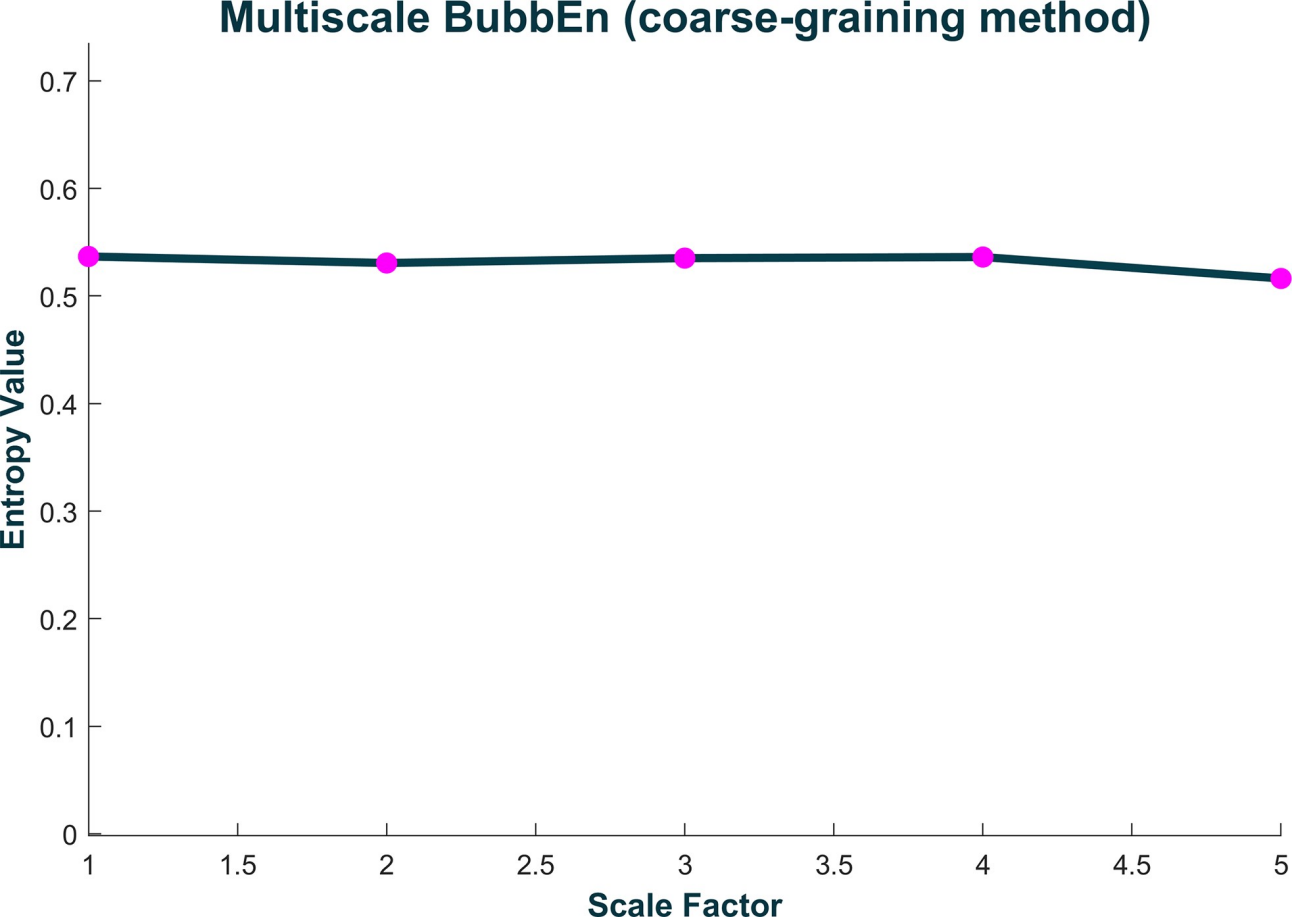
Representative plot of the multiscale entropy curve returned by any *Multiscale* or *Multiscale Cross* entropy function. The curve shown corresponds to multiscale bubble entropy of a Gaussian white noise signal (N = 5000, μ = 0, σ = 1), calculated over 5 coarse-grained time scales, with estimator parameters: embedding dimension (*m*) = 2, time delay (*τ*) = 1.

**Fig 2:**
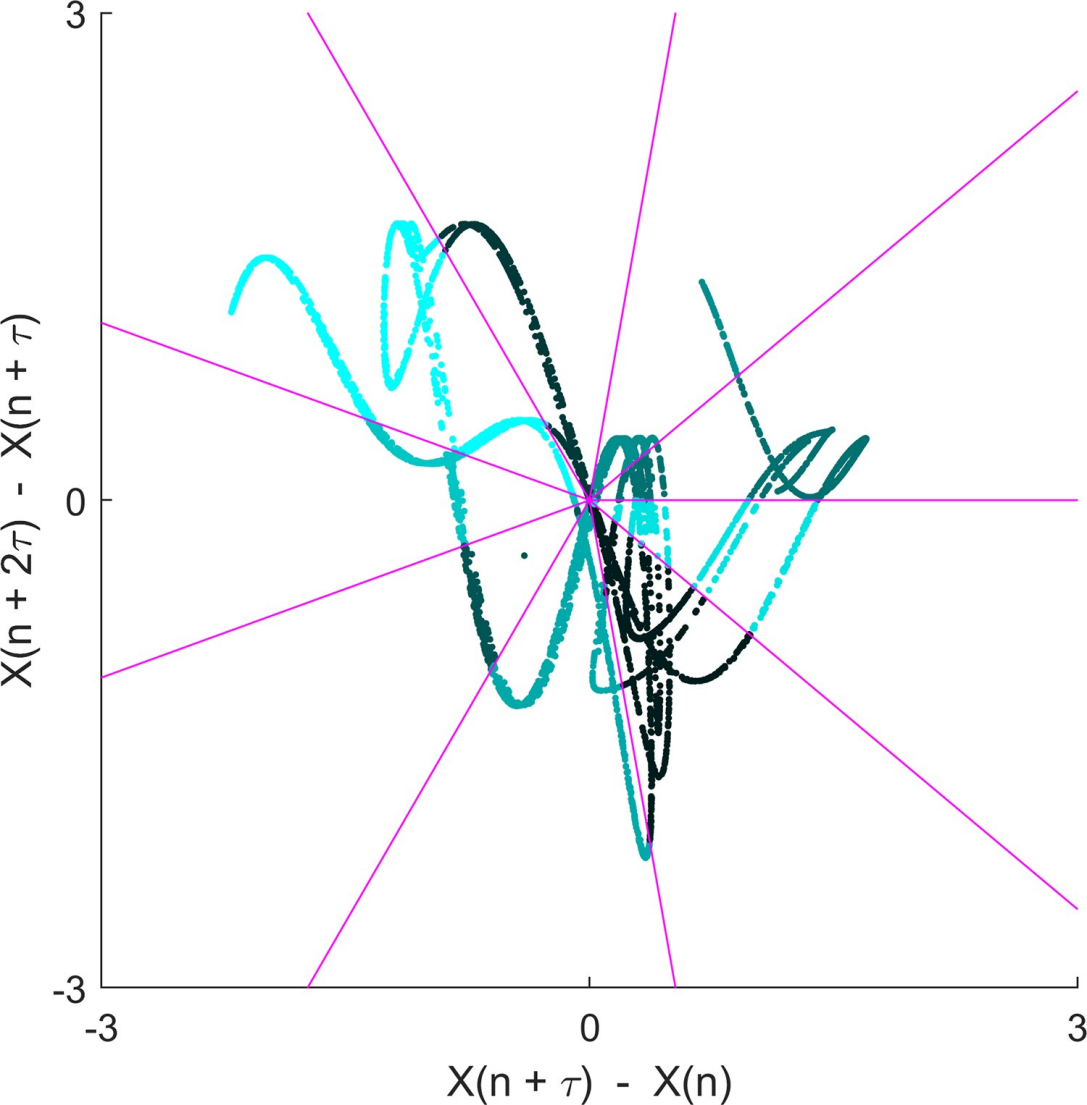
Second-order difference plot returned by the phase entropy function (PhasEn). Representative second-order difference plot of the x-component of the Henon set of equations (α = 1.4, β = 0.3), calculated with a time-delay (*τ*) = 2 and partitions (*K*) = 9.

**Fig 3:**
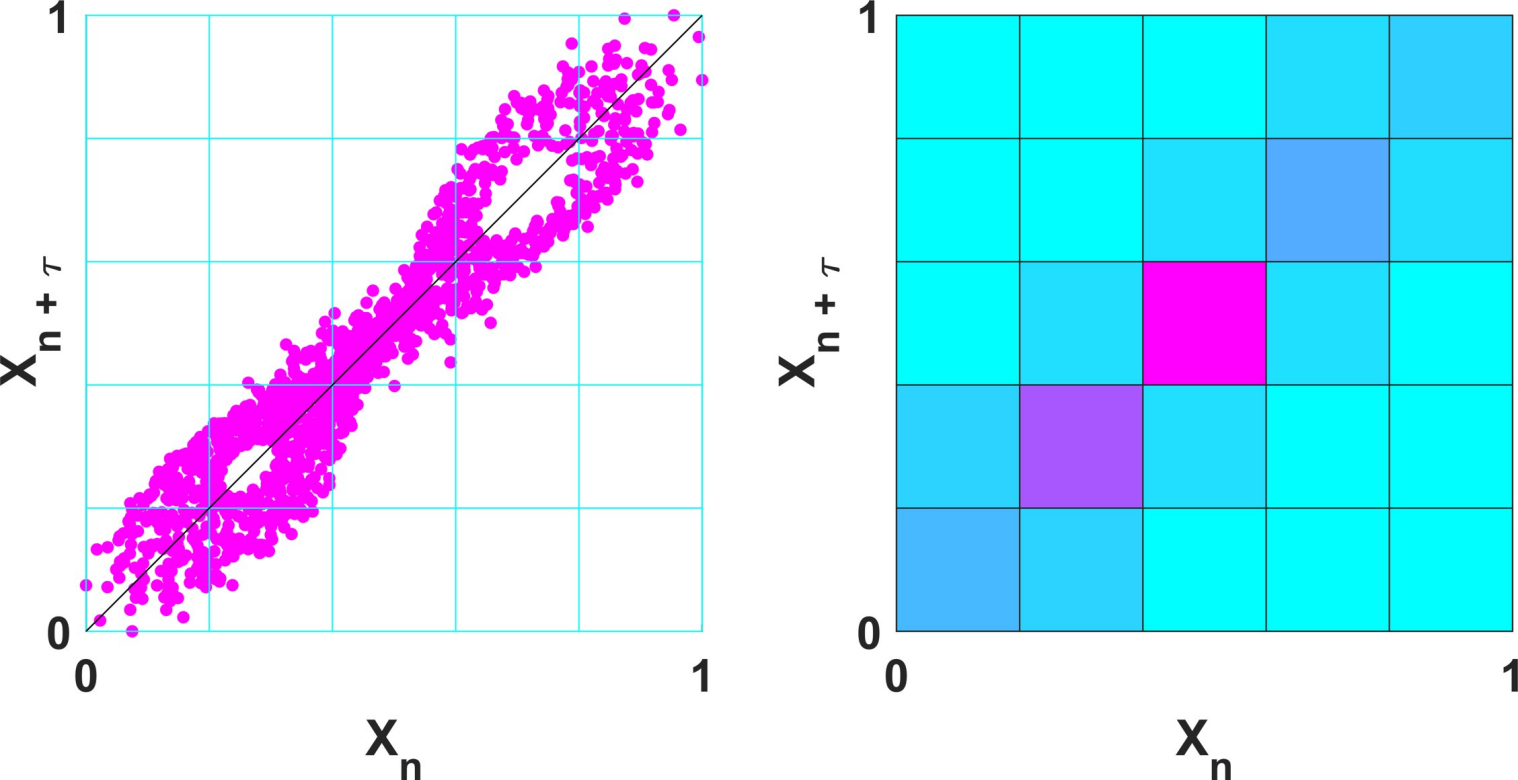
Poincaré plot and bivariate histogram returned by the gridded distribution entropy function (GridEn). Representative Pioncaré plot and bivariate histogram of the x-component of the Lorenz system of equations (σ = 10, β = 8/3, ρ = 28), calculated with grid partitions (*m*) = 5 and a time-delay (*τ*) = 2.

With certain *Base* and *Cross* functions, it is possible to calculate entropy using variant methods of the main estimator. For example, with the function for permutation entropy (PermEn) one can calculate the edge [65], weighted [70], amplitude-aware [11], modified [68], fine-grained [67], and uniform-quantization [71] permutation entropy variants, in addition to the original method introduced by Bandt and Pompe [66]. It is important to note that while the primary variable returned by each function is the estimated entropy value, most functions provide secondary and tertiary variables that may be of additional interest to the user. Some examples include the dispersion entropy function (DispEn) [8] which also returns the reverse dispersion entropy [50], the spectral entropy function (SpecEn) [74] which also returns the band-spectral entropy [102], and the Kolmogorov entropy function (K2En) [63] which also returns the correlation sum estimate. Furthermore, every *Multiscale* and *Multiscale Cross* function has the option to plot the multiscale (cross) entropy curve (Fig 1), as well as some *Base* functions which allow one to plot spatial representations of the original time series (Figs 2 and 3).

## Installation and dependencies

Major version releases of the EntropyHub toolkit can be directly installed through the native package repository for the MATLAB, Python and Julia programming environments. Beta development versions can be downloaded and installed from the directories of each programming language hosted on the EntropyHub GitHub repository– github.com/MattWillFlood/EntropyHub. EntropyHub is compatible with Windows, Mac and Linux operating systems.

### MATLAB

There are two additional toolboxes from the MATLAB product family that are required to experience the full functionality of the EntropyHub toolkit—the *Signal Processing Toolbox* and the *Statistics and Machine Learning Toolbox*. However, most functions will work without these toolboxes. EntropyHub is intended for use with MATLAB versions ≥ 2016a. In some cases, the toolkit may work on versions 2015a & 2015b, although it is not recommended to install on MATLAB versions older than 2016.

There are two ways to install EntropyHub in MATLAB.

**Option 1.** Note: Option 1 requires the user to be logged in to their MathWorks account.

1. In the MATLAB application, open the Add-Ons browser under the ‘Home’ tab by clicking ‘Get Add-Ons’ (S1A Fig).
2. In the search bar, search for “EntroypHub” (S1b Fig).
3. Open the resulting link and click ‘*add*’ in the top-right corner (S1c Fig).
4. Follow the instructions to install the toolbox (S1D Fig).

**Option 2**.

1. Go to the ‘EntropyHub–MatLab’ directory in the EntropyHub repository on GitHub (S1E Fig): https://github.com/MattWillFlood/EntropyHub/tree/main/EntropyHub%20-%20MatLab
2. Download the MATLAB toolbox file (*EntropyHub*.*mltbx*) file (S1F Fig).
3. Open the MATLAB application and change the current folder to the directory where the *EntropyHub*.*mltbx* file is saved (S1G Fig).
4. Double-click the *EntropyHub*.*mltbx* file to open it and click install (S1H Fig).

To check that EntropyHub has been correctly installed, enter “EntropyHub” at the command line and the EntropyHub logo should be displayed (S1I Fig).

### Python

There are several modules required to use EntropyHub in Python—*NumPy* [103], *SciPy* [104], *Matplotlib* [105], *PyEMD* [106], and *Requests*. These modules will be automatically installed alongside EntropyHub if not already installed. EntropyHub was designed using Python3 and thus is not intended for use with Python2 or Python versions < 3.6. EntropyHub Python functions are primarily built on top of the *NumPy* module for mathematical computation [103], so vector or matrix variables are returned as *NumPy* array objects.

There are 2 ways to install EntropyHub in Python. Option 1 is strongly recommended.

**Option 1. Note: Option 1 requires the ‘pip’ Python package installer**.

■ Using *pip*, enter the following at the command line (S2A Fig):
**        pip install EntropyHub**
  *Note: this command is case sensitive

**Option 2**.

1. Go to the ‘EntropyHub–Python’ directory in the EntropyHub repository on GitHub (S2B Fig): https://github.com/MattWillFlood/EntropyHub/tree/main/EntropyHub%20-%20Python
2. Download the *EntropyHub*.*x*.*x*.*x*.*tar*.*gz* folder and unzip it (S2C and S2D Fig).
3. Open a command prompt (**cmd** on Windows, **terminal** on Mac) or the Anaconda prompt if Anaconda is the user’s python package distribution (S2E Fig).
4. In the command prompt/terminal, navigate to the directory where the *EntropyHub*.*x*.*x*.*x*.*tar*.*gz* folder was saved and extracted (S2F Fig).
5. Enter the following in the command line (S2G Fig):
**        python setup.py install**
6. Ensure that an up-to-date version of the setuptools module is installed:
**        python -m pip install—upgrade setuptools**

To use EntropyHub, import the module with the following command (S2H Fig):

**import EntropyHub as EH**

To check that EntropyHub has been correctly installed and loaded, enter (S2H Fig):

**EH.greet()**

### Julia

There are a number of modules required to use EntropyHub in Julia—*DSP*, *FFTW*, *HTTP*, *DelimitedFiles*, *Random*, *Plots*, *StatsBase*, *StatsFuns*, *Statistics*, *GroupSlices*, *Combinatorics*, *Clustering*, *LinearAlgebra*, and *Dierckx* [45]. These modules will be automatically installed alongside EntropyHub if not already installed. EntropyHub was designed using Julia 1.5 and is intended for use with Julia versions ≥ 1.2.

To install EntropyHub in Julia,

1. In the Julia programming environment, open the package REPL by typing ‘]’ (S3A Fig).
2. At the command line, enter (S3B Fig):
**        add EntropyHub**
  *Note: this command is case sensitive.
  Alternatively, one can install EntropyHub from the EntropyHub.jl GitHub repository:
  **        add** **https://github.com/MattWillFlood/EntropyHub.jl**

To use EntropyHub, import the module with the following command (S3C Fig):

**using EntropyHub**

To check that EntropyHub has been correctly installed and loaded, type (S3D Fig):

**EntropyHub.greet()**

### Supporting documentation and help

To help users to get the most out of EntropyHub, extensive documentation has been developed to cover all aspects of the toolkit, www.EntropyHub.xyz/#documentation-help. Included in the documentation are:

■ Instructions for installation.
■ Thorough descriptions of the application programming interface (API) syntax–function names, keyword arguments, output values, etc.
■ References to the original source literature for each method.
■ Licensing and terms of use.
■ Examples of use.

Supporting documentation is available in various formats from the following sources.

### **www.EntropyHub.xyz**

The EntropyHub website, www.EntropyHub.xyz (also available at MattWillFlood.github.io/EntropyHub) is the primary source of information on the toolkit with dedicated sections to MATLAB, Python and Julia, as well as release updates and links to helpful internet resources.

### EntropyHub guide

The *EntropyHub Guide*.*pdf* is the toolkit user manual and can be downloaded from the documentation section of the EntropyHub website or from the EntropyHub GitHub repository. In addition to the information given on the website, the *EntropyHub Guide*.*pdf* document provides some extra material, such as plots of fuzzy functions used for fuzzy entropy (FuzzEn) calculation, or plots of symbolic mapping procedures used in dispersion (DispEn) or symbolic-dynamic entropy (SyDyEn).

### MATLAB help browser

Custom built documentation for the MATLAB edition of the toolkit is accessible through the MATLAB help browser after installation. Every function has its own help page featuring several examples of use ranging from basic to advanced. To access this documentation, open the help browser in the MATLAB application and at the bottom of the contents menu on the main page, under ‘Supplemental Software’, click on the link ‘EntropyHub Toolbox’.

### EntropyHub.jl

Custom documentation for the Julia edition of the toolkit can also be found at MattWillFlood.github.io/EntropyHub.jl (linked to the EntropyHub website). Following Julia package convention, the Julia edition is given the suffix ‘.jl’ and is hosted in a standalone GitHub repository linked to the main EntropyHub repository.

### Seeking further help

Within each programming environment, information about a specific function can be displayed in the command prompt by accessing the function docstrings. For example, to display information about the approximate entropy function (ApEn), type:

MATLAB:    help ApEn
Python:        help(EntropyHub.ApEn)        (if imported as EntropyHub)
Julia:        julia>?                 (to open help mode in the REPL)
                        help?> ApEn

#### Contact

For help with topics not addressed in the documentation, users can seek help by contacting the toolkit developers at help@entropyhub.xyz. Every effort will be made to promptly respond to all queries received.

To ensure that EntropyHub works as intended, with accurate and robust algorithms at its core, users are encouraged to report any potential bugs or errors discovered. The recommended way to report issues is to place an issue post under the ‘Issues’ tab on the EntropyHub GitHub repository. Doing so allows other users to find answers to common issues and contribute their own solutions. Alternatively, one can notify the package developers of any issues via email to fix@entropyhub.xyz.

Continuous integration of new and improved entropy methods into the toolkit is a core principle of the EntropyHub project. Thus, requests and suggestions for new features are welcomed, as are contributions and offers for collaboration. EntropyHub developers will work with collaborators to ensure that contributions are valid, translated into MATLAB, Python and Julia, and follow the formatting adopted throughout the toolkit. Please contact info@entropyhub.xyz regarding any proposals that wish to be made.

### Validation

**Fig 4:**
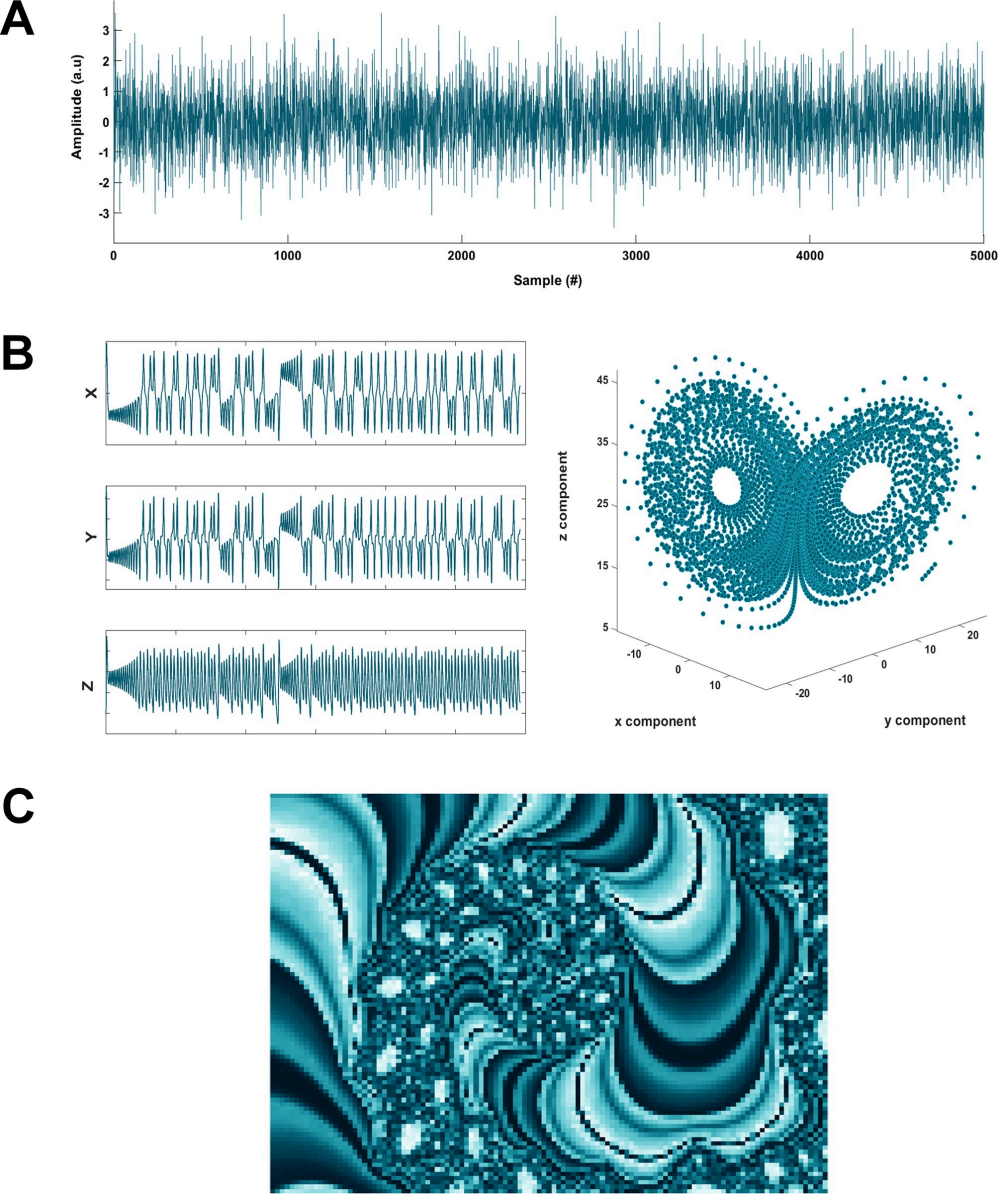
Sample datasets available with the EntropyHub toolkit through the ExampleData function. (a) A gaussian white noise time series, (b) the Lorenz system of equations, (c) a Mandelbrot fractal.

Included in EntropyHub are a number of sample time series and image datasets which can be used to test the validity of the toolkit functions (Fig 4). Included in these datasets are random number sequences (gaussian, uniform, random integers), chaotic attractors (Lorenz, Hénon), and matrix representations of images (Mandelbrot fractal, random numbers, etc.). Importing these datasets into the programming environment is done using the ExampleData function (Table 2), which requires an internet connection. Every example presented in the supporting documentation on the EntropyHub website, in the MATLAB help browser, or in the *EntropyHub Guide*.*pdf*, employs the same sample datasets provided by the ExampleData function. Therefore, users can replicate these examples verbatim to verify that the toolkit functions properly on their computer system. The following subsections demonstrate the implementation of several *Base*, *Cross-*, *Bidimensional*, *Multiscale* and *Multiscale Cross-*entropy methods as a proof-of-principle validation. Note: the examples in the following subsections use MATLAB syntax, but the implementation of these functions and the values they return are the same when using Python and Julia.

### *Base* entropy

A sequence of normally distributed random numbers (Fig 4A; *N* = 5000, mean = 0, SD = 1) is imported and approximate entropy is estimated using the default parameters (embedding dimension = 2, time delay = 1, threshold = 0.2*SD[X]).

>> X = ExampleData(‘gaussian’);

>> ApEn(X)

2.33505 2.29926 2.10113

Random number sequences produce high entropy values as such sequences possess maximum uncertainty or unpredicatbility. The high approximate entropy values (> 2) returned in this example, corresponding to estimates for embedding dimensions of 0, 1 and 2, are in the expected range for such time series.

### *Cross-*entropy

The *x*, *y* and *z* components of the Lorenz system of equations (Fig 4B; *N* = 5917, σ = 10, β = 8/3, ρ = 28, *x*_*0*_ = 10, *y*_0_ = 20, *z*_*0*_ = 10) are imported and cross-permutation entropy is estimated using the *x* and *y* components with the default parameters (embedding dimension = 3, time delay = 1).

>> X = ExampleData(‘lorenz’);

>> XPermEn(X(:,1:2))

0.17771

The Lorenz system is commonly employed in nonlinear dynamics as its attractor exhibits chaotic behaviour. Thus, the low cross-permutation entropy estimate returned here (0.1771) reflects the high degree of deterministic structure shared between the *x* and *y* components of the Lorenz system.

### *Bidimensional* entropy

A matrix of normally distributed (Gaussian) random numbers is imported (Fig 4C; *N* = 60x120, mean = 0, SD = 1) and bidimensional dispersion entropy is estimated with a template submatrix size of 5 and all other parameters set to default values (time delay = 1, number of symbols = 3, symbolic mapping transform = normal cumulative distribution function).

>> X = ExampleData(‘gaussian_Mat’);

>> DispEn2D(X, ‘m’, 5)

8.77894

The high value of the bidimensional dispersion entropy estimate corresponds to those previously reported for Gaussian white noise [83].

### *Multiscale* entropy

A chirp signal (*N* = 5000, t_0_ = 1, t_0_ = 4000, normalised instantaneous frequency at t_0_ = 0.01Hz, instantaneous frequency at t_1_ = 0.025Hz) is imported and multiscale sample entropy is estimated over 5 coarse-grained temporal scale using the default parameters (embedding dimension = 2, time delay = 1, threshold = 0.2*SD[X]). Note: a multiscale entropy object (*Mobj*) must be used with multiscale entropy functions.

>> X = ExampleData(‘chirp’);

>> Mobj = MSobject(‘SampEn’);

>> MSEn(X, Mobj, ’Scales’, 5)

0.2738 0.3412 0.4257 0.5452 0.6759

The chirp signal imported in this example represents a swept-frequency cosine with a linearly decreasing period length. The coarse-graining procedure of multiscale entropy [15] functions as a low-pass filter of the original time series, with a lower cut-off frequency at each increasing time scale. Therefore, the coarse-graining procedure increasingly diminishes the localised auto-correlation of the chirp signal at each temporal scale, increasing the entropy. This reflects the increasing sample entropy values from low (0.2738) to moderate (0.6759) returned by the *MSEn* function.

### *Multiscale cross*-entropy

Two sequences of uniformly distributed random numbers (*N* = 4096, range = [0, 1]) are imported and multiscale cross-distribution entropy is estimated over 7 coarse-grained temporal scales with the default parameters (embedding dimension = 2, time delay = 1, histogram binning method = ‘sturges’, normalisation with respect to number of histogram bins = true).

>> X = ExampleData(‘uniform2’);

>> Mobj = MSobject(‘XDistEn’);

>> XMSEn(X, Mobj)

0.95735 0.86769 0.83544 0.80433 0.82617 0.77619 0.78893

As expected, the *normalised* multiscale cross-distribution entropy values remain relatively constant over multiple time scales as no information can be gained about one sequence from the other at any time scale.

## Discussion

The growing number of entropy methods reported in the scientific literature for time series and image analysis warrants new software tools that enable researchers to apply such methods [2, 3, 38]. Currently, there is a dearth of validated, open-source tools that implement a comprehensive array of entropy methods at the command-line with options to modify multiple parameter values. EntropyHub is the first toolkit to provide this functionality in a package that is available in three programming languages (MATLAB, Python, and Julia) with consistent syntax, and is supported by extensive documentation (Table 3). To the best of the Authors knowledge, EntropyHub is also the first toolkit to provide multiple functions for bidimensional entropy [82–86], multiscale entropy [14, 15, 35, 90, 96] and multiscale cross-entropy analyses [40, 97, 98] all in one package. Specific programming language editions of the EntropyHub toolkit are hosted on the native package repositories for MATLAB, Python and Julia (Table 3), facilitating straightforward installation and version updates. EntropyHub is compatible with both Windows, Mac and Linux operating systems, and is open for use under the Apache License (Version 2.0) on condition that the present manuscript be cited in any outputs achieved through the use of the toolkit.

**Table 3:** List of resources for the EntropyHub toolkit.

**Online Resources**	
EntropyHub Website	www.EntropyHub.xyz
	MattWillFlood.github.io/EntropyHub
GitHub Repository	www.github.com/MattWillFlood/EntropyHub
	www.github.com/MattWillFlood/EntropyHub.jl (*Julia only repository*)
MATLAB Package	www.mathworks.com/matlabcentral/fileexchange/94185-entropyhub
Python Package	pypi.org/project/EntropyHub/
Julia Package	juliahub.com/ui/Packages/EntropyHub/npy5E/0.1.0
**Contact Details**	
General Inquiries	info@entropyhub.xyz
Help and Support	help@entropyhub.xyz
Reporting Bugs	fix@entropyhub.xyz

All information about the toolkit, including installations instructions, documentation, and release updates can be found on the main EntropyHub website. Users can get in touch directly with the package developers by contacting the email addresses provided.

The application of entropy in the study of time series data is becoming more common in all manner of research fields such as engineering [17, 18], medicine [19–23] and finance [24–27]. The broad range of entropy functions provided by EntropyHub in multiple programming languages can serve to support researchers in these fields by characterising the uncertainty and complexity of time series data with various stochastic, time-frequency and chaotic properties. Additionally, this is the first toolkit to provide several functions for performing bidimensional (2D) entropy analysis, which can enable users to estimate the entropy of images and matrix data.

The goal of EntropyHub is to continually integrate newly developed entropy methods and serve as a cohesive computing resource for all entropy-based analysis, independent of the application or research field. To achieve this goal, suggestions for new features and contributions from other researchers are welcomed.

## Supporting information

S1 Fig Instructions for installing EntropyHub in MATLAB. (TIF)

S2 Fig Instructions for installing EntropyHub in Python. (TIF)

S3 Fig Instructions for installing EntropyHub in Julia. (TIF)
