# Supplementary figures and images for: EntropyHub: An open-source toolkit for entropic time series analysis
Source: PLoS One. 2021 Nov 4;16(11):e0259448. doi: 10.1371/journal.pone.0259448 (PMC8568273; doi:10.1371/journal.pone.0259448)

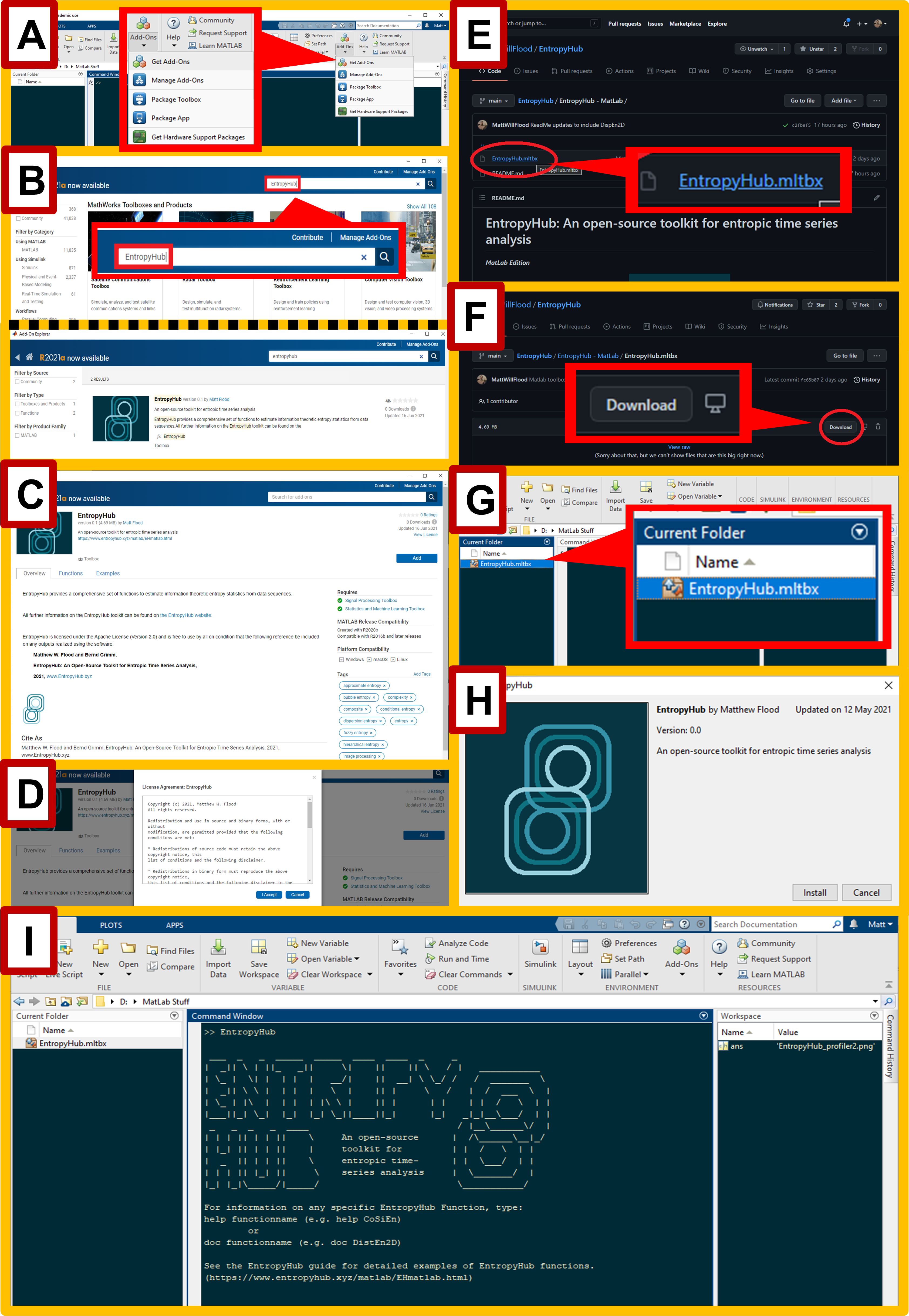

Supplement: S1 Fig — (TIF) [file pone.0259448.s001.tif]

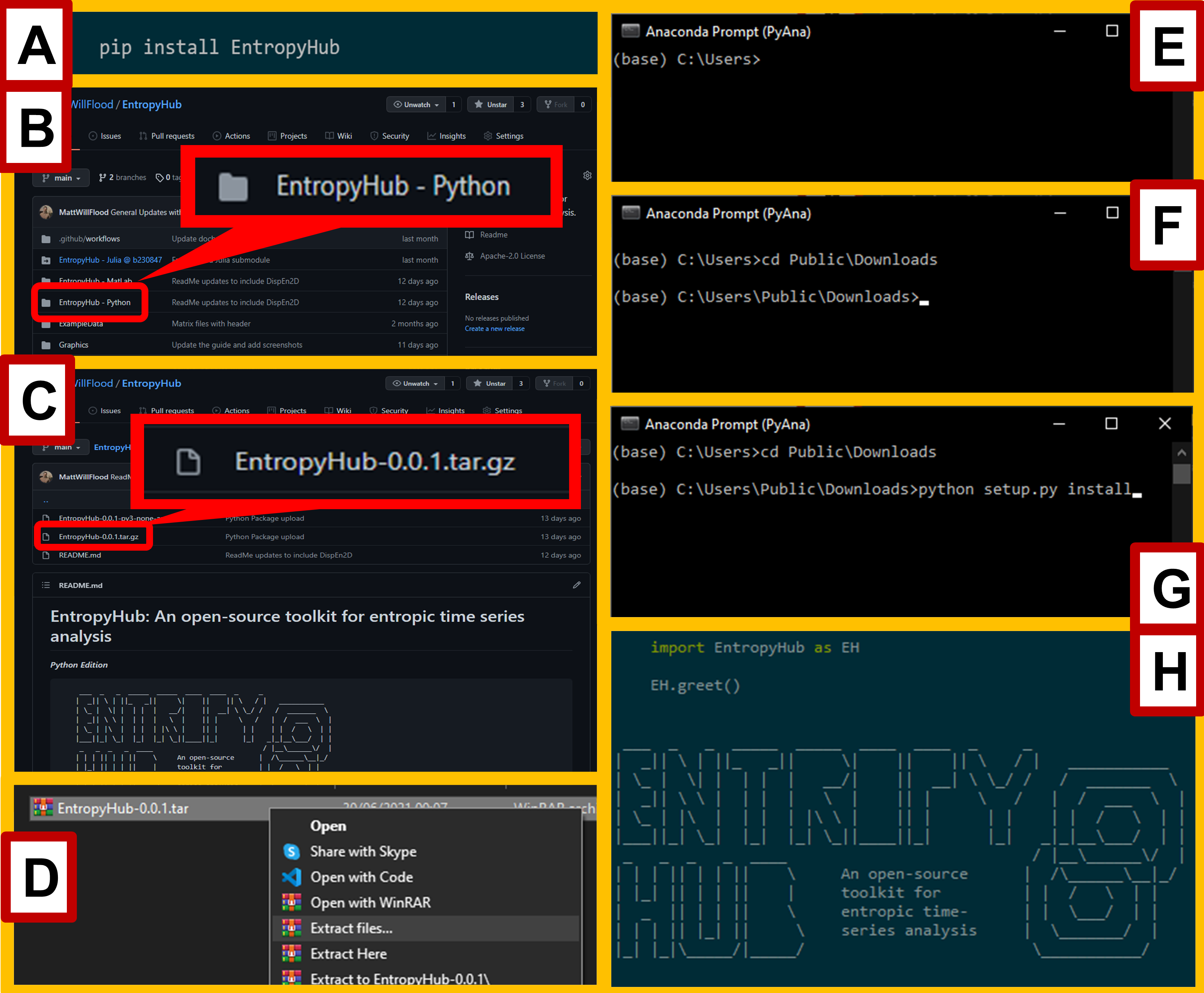

Supplement: S2 Fig — (TIF) [file pone.0259448.s002.tif]

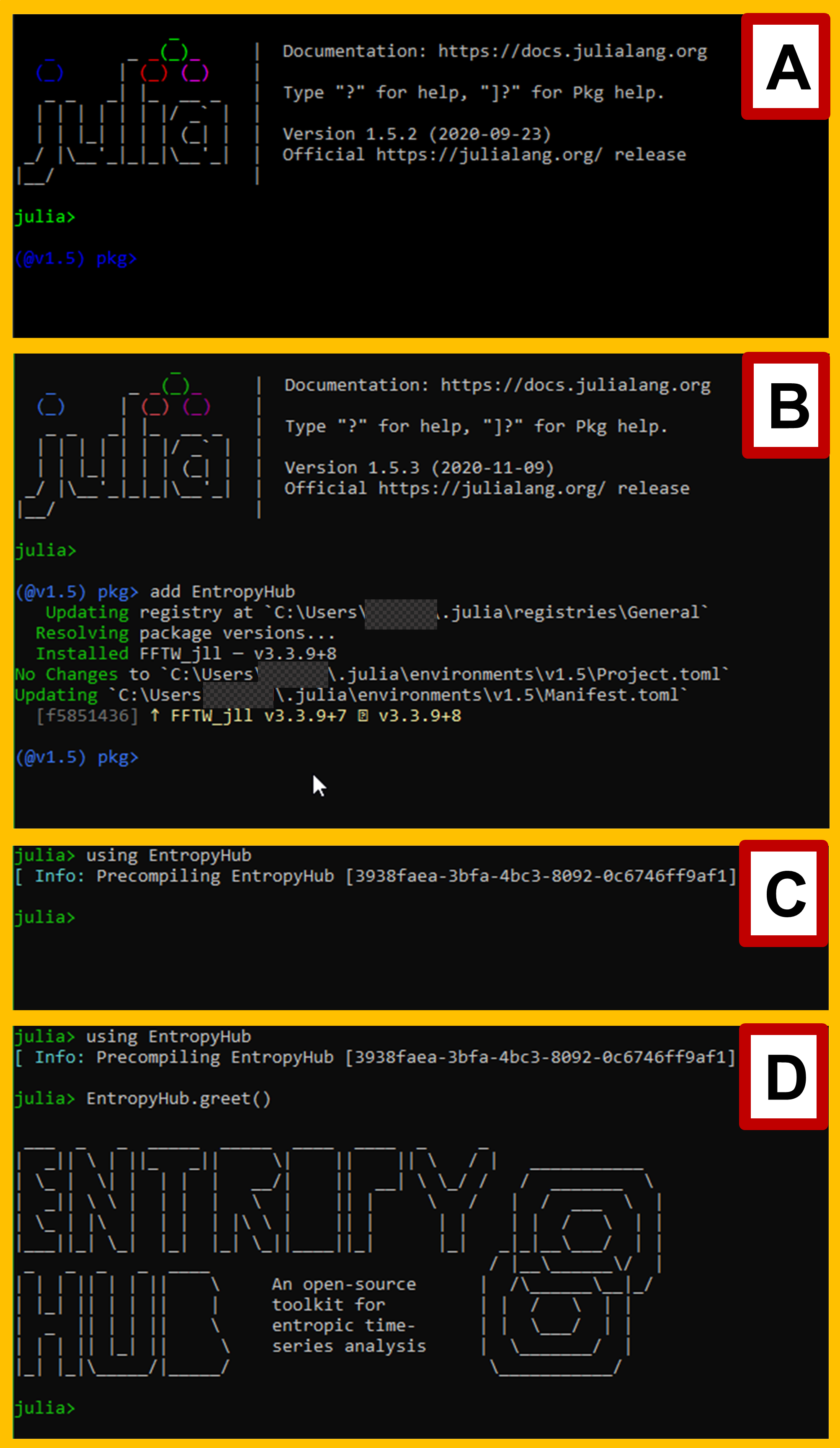

Supplement: S3 Fig — (TIF) [file pone.0259448.s003.tif]
